# Exploiting *Brassica rapa* L. subsp. *pekinensis* Genome Research

**DOI:** 10.3390/plants13192823

**Published:** 2024-10-09

**Authors:** Faujiah Nurhasanah Ritonga, Zeyu Gong, Yihui Zhang, Fengde Wang, Jianwei Gao, Cheng Li, Jingjuan Li

**Affiliations:** 1 Shandong Key Laboratory of Bulk Open-field Vegetable Breeding, Ministry of Agriculture and Rural Affairs Key Laboratory of Huang Huai Protected Horticulture Engineering, Institute of Vegetables, Shandong Academy of Agricultural Sciences, Jinan 250100, China; faujiahnurhasanah@usu.ac.id (F.N.R.); zyh_0923@163.com (Y.Z.); wfengde@163.com (F.W.); jwg_738@163.com (J.G.); 2 Faculty of Forestry, Universitas Sumatera Utara, USU 2 Bekala Campus, Pancurbatu, Deli Serdang 20355, Indonesia; 3 Shandong Provincial Key Laboratory of Plant Stress, College of Life Science, Shandong Normal University, Jinan 250358, China; s_f_gzy@foxmail.com

**Keywords:** agricultural traits, Chinese cabbage, genome, omics, regulatory networks

## Abstract

Chinese cabbage, *Brassica rapa* L. subsp. *pekinensis* is a crucial and extensively consumed vegetable in the world, especially Eastern Asia. The market demand for this leafy vegetable increases year by year, resulting in multiple challenges for agricultural researchers worldwide. Multi-omic approaches and the integration of functional genomics helps us understand the relationships between Chinese cabbage genomes and phenotypes under specific physiological and environmental conditions. However, challenges exist in integrating multi-omics for the functional analysis of genes and for developing potential traits for Chinese cabbage improvement. However, the panomics platform allows for the integration of complex omics, enhancing our understanding of molecular regulator networks in Chinese cabbage agricultural traits. In addition, the agronomic features of Chinese cabbage are significantly impacted by the environment. The expression of these agricultural features is tightly regulated by a combination of signals from both the internal regulatory network and the external growth environment. To comprehend the molecular process of these characteristics, it is necessary to have a prior understanding of molecular breeding for the objective of enhancing quality. While the use of various approaches in Chinese cabbage is still in its early stages, recent research has shown that it has the potential to uncover new regulators both rapidly and effectively, leading to updated regulatory networks. In addition, the utilization of the efficient transformation technique in conjunction with gene editing using CRISPR/Cas9 will result in a reduction in time requirements and facilitate a more precise understanding of the role of the regulators. Numerous studies about Chinese cabbage have been conducted in the past two decades, but a comprehensive review about its genome still limited. This review provides a concise summary of the latest discoveries in genomic research related to Brassica and explores the potential future developments for this species.

## 1. Introduction

The Brassica crop species’ are composed of three diploid species: *Brassica rapa* (AA), *Brassica nigra* (BB), and *Brassica oleracea* (CC). Additionally, there are three amphidiploid species: *Brassica juncea* (AABB), *Brassica napus* (AACC), and *Brassica carinata* (BBCC). These species collectively constitute the widely recognised ‘triangle of U’ paradigm [1]. *B. rapa* is regarded as a model plant for studying genome polyploidy in the context of evolutionary studies. Its genome is classified as AA. Previously, *Brassica rapa* L. was categorised as two separate species, namely *Brassica campestris* and *B. rapa*. These two species are capable of interbreeding. Regrettably, no molecular marker is currently available to differentiate between them. As a result, these two species have been merged into a single species, specifically *B. rapa*. *Brassica rapa* is cultivated globally as a significant vegetable and oilseed crop with commercial value. Due to frequent inter-species hybridization, nuclear genomes have undergone genetic recombination, resulting in the loss of species-specific DNA sequences [2].

*Brassica rapa* was discovered to exhibit a wide range of subspecies with distinct morphotypes, including turnip (subsp. *rapa*), yellow sarson (subsp. *trilocularis*) which is cultivated for seed oil, non-heading *B. rapa* (subsp. *chinensis*), and heading B. rapa (subsp. *pekinensis*) which is a leafy vegetable [3]. Brassica species have undergone a whole-genome triplication (WGT) event, which was absent in the model plant *Arabidopsis thaliana*. This event is crucial in the process of speciation and the development of different forms in Brassica plants [3,4]. WGT leads to an enlarged gene pool, which enables multicopy genes to develop distinct or novel roles, such as sub-functionalization or neofunctionalization [4].

Scientifically, Chinese cabbage, referred to as *Brassica rapa* L. subsp. *pekinensis*, is a crucial and extensively consumed vegetable in Eastern Asia [5]. Kimchi cabbage is a widely recognised term in Korea [6], but it is also referred to as napa cabbage [7,8], cool-weather Chinese cabbage [9], and headed Chinese cabbage [10]. Chinese cabbage is a type of vegetable that has large pale-green leaves and wide, white midribs, which come together to create a head [11]. Chinese cabbage possesses a rich nutritional profile, containing significant levels of antioxidants, phytochemicals, and mineral elements [12,13]. Additionally, it is known for its delightful flavour [12,13]. There is an increasing demand in the market for Chinese cabbage that possesses resilience to many types of stress, exhibits a delightful taste, has an attractive appearance, and is cost-effective. This has put significant pressure on agricultural researchers worldwide to develop high-quality varieties that meet these criteria [7].

Climate change has led to increased weather patterns, such as droughts, floods, and extreme temperatures, which are expected to cause huge crop losses and threaten worldwide food stability [7]. Pathogens are also a main cause of defective traits in plants. To improve food tolerance in biotic and abiotic environments, strategies have been developed to improve plant tolerance in *B. rapa* [6]. Introgression breeding and interspecific hybridization are widely used methods for genetic improvement, such as transferring clubroot tolerance from Chinese cabbage to canola [14] and vice versa [15]. These techniques enhance genetic diversity and improve the agronomic performance of novel cultivars. Inter-specific hybridization [16] and back-crossing [17] are also powerful tools for transferring favourable genes between species in plant breeding programs.

**Figure 1:**
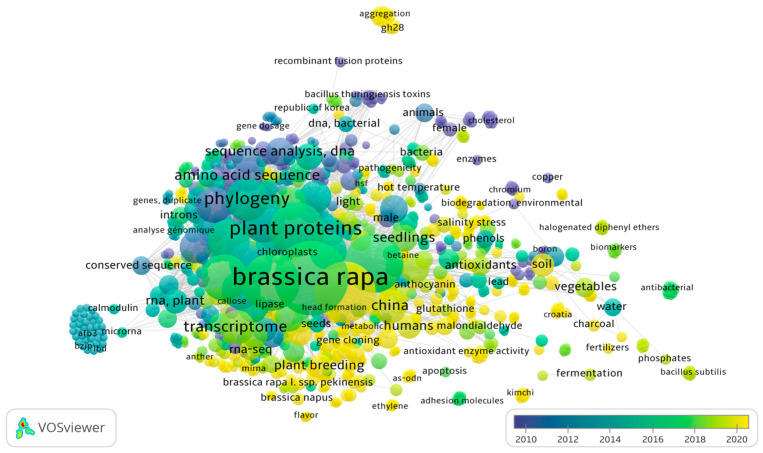
Term co-occurrence map of Chinese cabbage studies using VOSviewer software version 1.620 (https://www.vosviewer.com/). VOSviewer analysed text mining and bibliometric of scientific papers by observing the outputs of term (keyword) co-occurrence analysis. Studies about Chinese cabbage from 2010–2020 relate to biotic and abiotic stresses, head formation, and multi-omics (accessed: 30 September 2024).

For nearly 100 years, traditional breeding has been successful for preventing starvation but has stagnated due to long breeding times and limited crossbreeding crops. A molecular-based selection strategy has emerged, replacing phenotype-based breeding. This has led to the widespread use of quantitative trait locus (QTL) and Marker Assisted Selection (MAS) in Chinese cabbage studies, as well as the potential application of Single Nucleotide Polymorphism (SNP) and InDel markers [18,19]. Before 2016, Chinese cabbage studies were primarily conducted in South Korea [20,21,22], but recent research has expanded to other countries, including China, and countries in Europe (Figure 1) [23,24,25]. In Figure 1, we present the co-occurrence map of Chinese cabbage studies to illustrate the updated studies of Chinese cabbage. This review elucidates the advanced genome research in Chinese cabbage and provides insights into the past, present, and future of Chinese cabbage breeding.

## 2. The Field of Multi-Omics Technologies in Chinese Cabbage

Several intriguing omics technologies have arisen in recent decades. An essential stage in evaluating genome research of Chinese cabbage is to identify potential genes that are still limited to clarify the genetic basis of complex features in Chinese cabbage. In the past, this attempt encountered substantial obstacles because of a lack of relevant publications resulting from the limited accessibility of this research compared to other crops. An integrated panomics platform enables the incorporation of intricate omics to develop models for predicting complex characteristics. Integrating systems biology with multi-omics datasets can augment our comprehension of molecular regulator networks for Chinese cabbage management.

The omics-based methodologies have demonstrated their value in investigating the genetic and molecular mechanisms underlying crop development, specifically concerning alterations in mineral nutrients, metabolites, proteins, transcript levels, and DNA [26]. The introduction of next-generation sequencing (NGS) technologies has resulted in the efficient and quick production of large amounts of data for genomes, epigenomes, transcriptomes, proteomes, metabolomes, and phenomes [27]. This data can provide insights into gene functions and networks in the context of environmental and physiological stress responses [28]. To gather comprehensive information on the genomic data of Chinese cabbage, we extensively reviewed all relevant publications from various journals and publishers such as Elsevier, Springer Nature, MDPI, Frontiers, Wiley Online Library, Taylor and Francis, and others.

In this review, we present a comprehensive overview of the latest developments in omics investigations focused on Chinese cabbage.

### 2.1. Genomics

Genomics is the field of research that examines genes and genomes. It specifically investigates the structure, function, evolution, mapping, epigenomic, mutagenomic, and genome editing aspects [29]. Structural genomics is a genomics branch that focuses on identifying and utilising molecular markers in tagging and mapping genes of interest. These markers are then used in plant breeding programmes. There are two approaches used in structural genomics, namely PCR and non-PCR-based techniques. Unfortunately, the use of non-PCR-based approaches is restricted in Chinese cabbage investigations. In contrast, PCR-based approaches have been performed in Chinese cabbage for several decades [30,31].

Before multi-omics investigations, a PCR-based technique was used to detect certain traits of Chinese cabbage, such as microsatellite markers [30], anchor single sequence repeats (SSRs), Sequence-related Amplified Polymorphism (SRAP) [32,33], and single nucleotide polymorphisms (SNPs) [34]. The SNPs are genetic differences that occur at a single nucleotide position in the organism’s genome. SNP analysis can be conducted by sequencing genomic PCR results from diverse individuals. A new Chinese cabbage map was constructed using 11 newly discovered polymorphic markers, in addition to 227 previously mapped marker loci. The updated map now has a total of 238 marker loci [30].

Microarray technology is used in plant molecular breeding to measure gene expression and detect specific DNA sequences, including SNPs. It involves binding thousands to millions of recognized nucleic acid fragments to a chip, that is then immersed with either DNA or RNA. This technique has been used to observe genes related to hormones and predict hybrid performance in Chinese cabbage. By using microarray analysis, it was a total of 4646 genes showed different expressions, following floral bud size which was related to hormones [35]. Furthermore, a heterotic prediction has been performed in headed Chinese cabbage to enhance breeding efficiency. This prediction is based on whole genome SNP markers and the phenotypic of parental inbred lines to determine hybrid performance [36].

Derived cleaved amplified polymorphic sequence (dCAPS) detects single base changes, but SNPs are unsuitable for CAPS markers. Kompetitive Allele-Specific PCR (KASP) marker genotyping technology allows accurate bi-allelic scoring of SNPs and specific loci across genomic DNA samples. KASP genotyping, combined with dCAPS, detects the retr02 allele in Chinese cabbage accessions. KASP is flexible, affordable, and efficient, making it beneficial for marker-assisted selection [37]. The study found that a single recessive nuclear gene, BraA09g066480.3C, controls the wax deficiency mutant cer1 phenotype in Chinese cabbage leaves. Using MutMap and KASP analysis, an SNP in the fourth exon of BraA09g066480.3C was assumed to cause a substitution of amino acid, resulting in protein changes [38].

The advent of NGS enabled the identification and usage of SNPs [34]. Eight gene-specific and gene-flanking markers for glucosinolates (GSLs) were identified in a combination of SSRs and recombinant inbred lines (RILs) derived from a cross between yellow sarson and Chinese cabbage. Additionally, 148 SSR markers were included in the preexisting ultra-dense genetic map of Chinese cabbage. The genetic map was utilised to conduct QTL mapping for GSLs, with the identification of loci responsible for the production of GSLs achieved by the application of gene-specific markers [33].

The first reference genome for Brassica species, Chiifu v1.5, was achieved using Chinese cabbage [29]. *B. rapa* Chiifu variant which originated from temperate regions, is more commonly used as a database source than *B. rapa* Kenshin which is from subtropical and tropical regions [39]. Sequencing technologies have led to the creation of two improved genome assembly versions of Chinese cabbage (Chiifu v2.0, v2.5, and v3.0) and an updated genome annotation version, namely Chiifu v3.5 [29,40,41,42]. Chiifu v1.5 and v2.5 were assembled using Illumina short reads, Chiifu v3.0 was generated using PacBio (Menlo Park, CA, USA), optical maps, and Hi-C technologies [29,40], and Chiifu v3.5 was updated using full-length PacBio sequencing [41]. Despite the considerable genomic studies performed on B. rapa, there is still a major absence of a complete genome annotation that encompasses gene structure, alternative splicing (AS) events, and non-coding genes in Chiifu v1.5, Chifuu v2.0, Chiifu v2.5, and Chifuu v3.0. Hence, Zhang, et al. [43] employed the single-molecular long-read method developed by PacBio to enhance gene models and generate the annotated genome version 3.5. The total number of full-length non-chimeric (FLNC) reads acquired was 753,041. These readings were then consolidated into 92,810 nonredundant consensus isoforms, which encompassed 48% of the genes that were annotated in the B. rapa reference genome annotation v3.1.

A study examined the effects of whole-genome triplication (WGT) on the variation within a species. This was completed by analysing the pan-genome of 16 newly assembled genomes and 2 previously published genomes using Illumina and PacBio sequencing technologies [43]. The availability of Chinese cabbage genome assemblies has been highly beneficial in comparative genomics and genetic breeding investigations of Brassica species.

Advances in sequencing technologies, like PacBio and Oxford Nanopore Technology (ONT), have revolutionized obtaining chromosome sequences, but the missing hundred sequences and thousand gaps still need to be minimized [3]. In addition, the Chinese cabbage Chiifu genome sequence enables the analysis of genome-wide identification, allowing for an understanding of multiple traits in Chinese cabbage. Several genome-wide identifications have been conducted in Chinese cabbage, namely TIR-NBS-LRR genes under TuMV infection [44], NRTs gene under low nitrate condition [45], microRNAs under *Plasmodiophora brassicae* infection [46], MYB transcription factors (TFs) in response to stresses in the male organ development stage [39], fork head-associated (FHA) for male sterility [47], expression and functional DELLA [48], and callose enzyme families [49].

Still, the demand for abiotic or biotic-resistant cultivars of Chinese cabbage is increasing, but traditional breeding methods are slow due to environmental conditions. The development of molecular biology techniques, particularly molecular markers, allows for the study of QTL and MAS, an effective breeding method [32]. To implement MAS, it is necessary to identify the specific gene responsible for a trait or the genetic region associated with that gene, which can be used for selective breeding purposes [11]. SSR markers together with MAS have been used in Chinese cabbage studies to improve quality, such as clubroot resistance in canola. This approach allows for the transfer of resistance genes to other cruciferous crops [14].

NGS-based bulked segregant analysis (BSA) is a valuable technique for identifying and mapping genes, particularly in crop breeding [50]. BSA is also known as a QTL mapping technique. It involves crossing a mutant to a wild type (WT) and identifying the mutants in the backcross population. This allows for rapid detection of increased homozygosity in a chromosomal region associated with the present phenotype of mutant [51]. Tipburn is a significant physiological disorder, causing 20% of phenotypic variance. Until now, most of the causes of tipburn cannot be successfully controlled by management precautions, suggesting a need for breeding tipburn resistant cultivars as the best long-term strategy in Chinese cabbage. The study identifies loci associated with tipburn resistance in the F2 population and the DH100 population using BSA and QTL mapping. Results indicate multiple genes contribute to tipburn disease resistance, with transgressive segregation patterns. High-quality bases were obtained from the DNA bulks of the tipburn susceptible and resistant populations, with 2,604,351 and 2,590,679 SNPs identified, respectively. It was also stated that the QTL gqbTRA06, containing *BrCRT2*, contributes to this issue where 51-bp deletion in the tipburn-susceptible line [51]. Breeding for tipburn resistance is the best long-term strategy. QTL has been studied for other traits like flowering time [18], yellow inner leaves [32], seed glucosinolates [33], leafy head formation [19], pathogen [10], and glossiness [52]. Representative breeding techniques are presented in Table 1. Chinese cabbage has a higher diversity of gene analogues than *A. thaliana*, with some domain genes being more abundant [53]. Despite the availability of high-quality genome sequences, studies about gene functions remain challenging [54]. A pangenome of Chinese cabbage was assembled to describe resistance gene analogues (RGAs), with significant conservation (93%) between the *B. rapa* and *B. napus*. It was found that presence–absence variation (PAV) affected 309 RGAs. Furthermore, a total of 223 missed RGAs were found in the reference genome. A total of 138 candidate RGAs were found within the recognized Chinese cabbage disease resistance QTL, with most under negative selection [10]. Whole-genome resequencing together with BSR-Seq and linkage analysis were also used to map and predict *BrCAO*, a gene that encodes chlorophyllide an oxygenase, a recessive nuclear gene causing a pale green mutant in Chinese cabbage [55].

Functional genomics and mutagenomics are used to study structural genomics and gene function. Authentic genome engineering is being used to improve crops, such as Chinese cabbage breeding, using tools like the CRISPR/Cas9 system [60] and transcription activator-like effector nuclease (TALEN) [61]. The CRISPR system allows for the induction of precisely targeted mutations in a plant genome using a binary construct commonly delivered into the *Agrobacterium tumefaciens* strain GV3101 [62]. Recently, CRISPR/Cas9 has begun to be widely used in Chinese cabbage breeding (Table 2) [57]. This system allows for targeted mutations in plant genomes, with studies showing high indel efficiency in flowering time [60,63,64]. A study found that *BraFLC2* and *BraFLC3* double knockout lines in the Kenshin background showed 97.7% and 100% indel efficiency, respectively, in the T0 generation [63]. Furthermore, high-quality protoplasts from leaf mesophylls are required for genome editing research [13]. Particle bombardment is also used for efficient genetic transformation of isolated microspores in Chinese cabbage, but low efficiency is a key issue affecting functional verification [65].

Chinese cabbage, a major crop in Asia, produces over 50 megatons annually, accounting for 70% of the world’s yield. The diploid genome has complex genetic control, making genetic and genomic analysis challenging. Mutagenesis is crucial for functional genomics studies and breeding materials, but fully characterized mutant resources are lacking [68]. EMS mutagenesis is a crucial breeding and genomic research tool, resulting in mutants with heritable morphological variations in leaf colour, shape, head, bolting, and fertility. Seed EMS mutagenesis generates large-scale mutant libraries, while microspore EMS mutagenesis facilitates rapid homozygous mutants [69]. Chinese cabbage mutants have been obtained using EMS mutagenesis including a male sterile mutant [70], non-heading flat leaves mutant [71], heading compact and wrinkled leaves mutant, and so on. Map-based cloning is often used to identify mutant causal genes involved in biosynthesis, such as the *BrWAX2* gene in cuticular wax biosynthesis [70], *BrCER60.A09* in cuticular wax biosynthesis [72], and OCTOPUS (*BrOPS*) gene in leafy head development [73]. Recently, a study combined MutMap, KASP genotyping, and map-based cloning to recognize a causal gene that is involved in GA biosynthesis and related to leafy head formation, namely *BraA09g001440.3C* [74].

### 2.2. Transcriptomics

NGS technology, including small RNA, degradome, and transcriptome sequencing, has been successfully applied to Chinese cabbage to identify miRNAs and their targets [46]. This technology allows for the resolution of relationships among subspecies and domestication history. A huge number of SNPs from across the nuclear genome can be used for resolving the relationships and genetic structure of Chinese cabbage [75]. Reference Qi, et al. [75] identified SNPs obtained from 126 accessions worldwide utilizing high-throughput transcriptome data. Extensive examination of over 31,000 single nucleotide polymorphisms (SNPs) in the *B. rapa* genome provided proof of five separate genetic groups and confirmed that *B. rapa* crops originated in Europe and Central Asia. It confirmed the well-established South Asian and East Asian *B. rapa* groupings and provided evidence that pak choi, Chinese cabbage, and yellow sarson are likely monophyletic groups. Conversely, the oil-type B. rapa subsp. oleifera and brown sarson, exhibited polyphyllenism. *B. rapa* was introduced to Asia approximately 2400 to 4100 years ago while Chinese cabbage originated some 1200 to 2100 years ago through the hybridization of pak choi and B. rapa from European-Central Asia. Substantial differences in the levels of founder effects were deduced among the several subspecies of *B. rapa*. Extant historical records documenting these crops are consistent with deductions.

The implementation of extensive transcriptome on long-time scales is conducted to encompass the primary stages of vegetative growth, including heading and non-heading [76]. A comparable investigation was undertaken to ascertain the causative genes in transcriptome analysis of leaf size differential [77]. Multiple studies utilising transcriptomic data have been published on various aspects of Chinese cabbage. These include investigations on yellow leafy head development [78], the transition that initiated the leaf heading between cabbage and Chinese cabbage [76], the colour difference between white and yellow Chinese cabbage inner-leaf [79], clubroot-resistant and clubroot-susceptible genotypes against *P. brassicae* infection [80], and leaf size [77].

Transcriptome analysis and SSRs have been used to identify candidate genes in Chinese cabbage, with 41 candidate genes identified across 10 chromosomes. Additionally, 341 SSRs were detected in 5 replicated types, namely single, double, triple, quintuple, and sextuple nucleotide replications [31]. Studies have also combined metabolomics and transcriptomics data to identify differentially expressed metabolites (DEMs) and differentially expressed genes (DEGs) and the correlation between DEMs and DEGs was might consistent or not consistent. The KEGG analysis is employed to validate the findings and identify multiple co-regulated pathways [81]. A study using the Illumina RNA-seq platform analysed transcriptome profiling in spring-sown Chinese cabbage. and the results were confirmed by RT-qPCR. KEGG pathway analysis revealed that there was an enrichment in pathways related to sucrose and starch metabolism, hormones, and signal transduction, suggesting potential trait candidates [82]. Moreover, a global transcriptome profiling of roots of clubroot-susceptible and clubroot-resistant Chinese cabbage revealed different gene expression in defence, transport, metabolism, and signal transduction, with 151 genes presenting different expression patterns between the two near-isogenic lines (NILs) [80].

Transcriptome studies in Chinese cabbage revealed the involvement of some TFs under stresses [83], glucosinolate pathways [84], and leafy head formation [78]. Chinese cabbage studies have identified MYB as the most prevalent TF family. Recently, *MYC2/3/4*, which are basic helix–loop–helix (bHLH) TFs, have been found to play a role in Chinese cabbage secondary metabolites. Additionally, *MYC2/3/4* activates the involvement of genes in various aspects of plant development. In particular, *BrMYC2* has been shown to reduce the length of roots and hypocotyls, as well as seed length and weight. It also promotes bolting time and significantly increases the total GS accumulation to confer the optimum resistance in response to the pathogenic fungus, *Sclerotinia sclerotiorum* [85]. The identified TFs in Chinese cabbage are presented in Table 3.

### 2.3. Proteomics

Molecular proteomics is a technique employed to examine the total protein composition within an organism [88]. Proteomic analyses yield a substantial collection of data pertaining to the individual proteins implicated in particular biological reactions. Proteomic analysis has been employed in the study of Brassicaceae, specifically focusing on the processes of transmission of turnip mosaic virus over long distances in Brassica rapa L. ssp. Chinensis [89], fertility conversion in the thermo-sensitive male sterile line PK3-12S of winter rapeseed [90], and so on. Nevertheless, there is a scarcity of proteomic research particularly focused on Chinese cabbage. Highly sophisticated methods like SDS-PAGE, two-dimensional gel electrophoresis (2-DE), and two-dimensional differential gel electrophoresis (2D-DIGE) were devised and employed in gel-based approaches for protein separation [91,92]. Proteins extracted from the roots of B. rapa cv. For identification using mass spectrometry, 742 seedlings were subjected to 2-DE analysis. MALDI/TOF/MS analysis and BLASTp searches revealed the identification of nine differentially expressed genes (DEPs) from the roots of Brassica rapa L cv. Infection of 742 seedlings with plasmodiophora brassicae was conducted. Notably, all proteomic studies on Chinese cabbage primarily focus on P. brassicae infection. DEPs 3038 and 3129 were identified as proteins associated with salicylic acid (SA)-mediated systemic acquired resistance (SAR), DEPs 3414 and 2338 were associated with jasmonic acid (JA)/ethylene (ET)-mediated induced systemic resistance (ISR), and DEP 4043 was found to be involved in both SAR and ISR [93].

Isobaric tags for relative and absolute quantitation (iTRAQ) analysis are sophisticated methods used in quantitative proteomic research to effectively elucidate certain protein mechanisms [94]. The iTRAQ-based proteomic data revealed distinctive physiological and molecular alterations in the pre-intermediate stages of infection from P. brassicae in both the infected and control Chinese cabbage lines. The resistant/susceptible lines were found to have a total of 5003 proteins with varying abundances, which could be measured using dipeptide or polypeptide segments. Drawing from the differential proteome analysis results and pertinent previously published data, it is probable that Chinese cabbage either protected or opposed P. brassicae, as evidenced by the observed harm caused by the activation of cysteine, methionine, and tyrosine metabolism. Susceptible lines exhibited upregulation of glutamine synthetase cytosolic isozymes 1–5 and Arginine decarboxylase 2, indicating the involvement of these proteins in the response to P. brassicae poisoning [94]. Similarly, iTRAQ analysis performed by [95] revealed differential expression of 295 proteins involved in energy and lipid metabolism, plant defence, cell wall modification, and hormone biosynthesis and signalling during the secondary phase of the Chinese cabbage–P. brassicae interaction. Notably, the upregulation of proteins related to brassinosteroids (BR) metabolism was confirmed. Specifically, cycloartenol synthase (CAS1) and cytochrome P450 51G1 (CYP51G1), which play a significant role in BR biosynthesis, were increased after inoculation. This indicates that these proteins contribute to the development of clubroot tissue [95]. The proteomic findings shed fresh light on the development of germplasm resistance to P. brassicae and give a genetic foundation for breeding to enhance Chinese cabbage.

### 2.4. Metabolomics

Metabolomics is a comprehensive study of metabolites involved in biological system processes. However, metabolite profiling and variations in Chinese cabbage are still limited. Metabolomics and NGS have helped predict an initial metabolic network from an organism’s genome sequence. Major biochemicals, such as GABA, acetate, and phenylalanine, differentiate cabbages grown in two regions. These differences suggest that environmental factors like climate and geology influence the levels of these metabolites in Chinese cabbage [20]. Similarly, a recent metabolomic study was performed using ultra-high-performance liquid chromatography–tandem mass spectrometry (UPLC-MS/MS) comparing two areas (Jiaozhou and Jinan). Chinese cabbage is rich in nutrients like amino acids, lipids, organic acids, phenolic acids, nucleotides, flavonoids, saccharides, glucosinolates, vitamins, and alcohols. Interestingly, some unique metabolites are recognized in Jiazhou and were absent in Jinan namely S-(Methyl)glutathione and nicotinic acid adenine dinucleotide. These two metabolite concentrations were elevated in Jiazhou, illustrating that they are Jiaozhou-specific metabolites of Chinese cabbage [96]. KEGG analysis showed that DAMs were significantly enriched in tryptophan metabolism and thiamine metabolism [96]. A study on Chinese cabbage in South Korea found that its taste variations depend on cultivation season, impacting kimchi production quality. The analysis revealed that glucose, fructose, as well as malic acid, exhibit a positive correlation with sweetness, while glucoerucin and glucobrassicin positively correlate with bitterness [97]. Compared to other cruciferous species, Chinese cabbage is the most sensitive in response to salt stress [98]. However, Chinese cabbage has been studied for its salt resistance, with several critical and potential metabolite candidates such as Coenzyme Q10, acetyl-CoA, pyruvic acid, nicotinic acid, and ATP that could reduce the salt stress level in the Chinese cabbage [81]. Qinghua, a cultivar with high salt tolerance, can improve photosynthetic fluorescence ability, promoting organic matter synthesis [99]. A study also explored the metabolic basis of soluble sugar and GSLs in white and yellow inner leaves of six Chinese cabbage cultivars. The genes *BraA05gAOP1*, *BraA04gAOP4*, *BraA03gHT7*, and *BraA01gHT4*, as well as the transcription factors *BraA01gCHR11* and *BraA07gSCL1* are responsible for the synthesis of glucosinolates and soluble sugars due to their higher relative expression in the yellow inner-leaf Chinese cabbage than that in the white inner-leaf Chinese cabbage based on qRT-PCR and transcriptome data. *BraA01gCHR11* and *BraA07gSCL1* may regulate soluble sugar and glucosinolate biosynthesis. The major metabolites found were aliphatic glucosinolate and two soluble sugars [79].

## 3. Explored Chinese Cabbage Traits

### 3.1. Glossy Traits in Chinese Cabbage

Plant cell walls maintain mechanical hardness and protect against damage from biotic and abiotic factors. Cuticular wax functions by covering the surface of the outer plant and plays a crucial role in protecting plants against stresses. Glossiness is an important quality-related trait of Chinese cabbage which is caused by abnormal cuticular wax accumulation [52]. The glossy phenotype is crucial for enhancing the genetic diversity of Chinese cabbage and developing vibrant green variants through breeding. Prior research has indicated that the glossy characteristics are a result of impaired wax biosynthesis [59,72], which exhibited a reduction in the overall levels of wax, alkanes, ketones, and alcohols in its leaves, whereas the concentration of aldehydes increased in comparison to the wild type.

A genetic study has revealed that *BrCER1* is the causative gene responsible for the glossy trait observed in the cer1 phenotype. This gene encodes aldehyde decarboxylase [38]. Furthermore, it has been discovered that *BrWAX2* is a potential gene associated with *CER1*. The glossy mutant lacks *CER1*. The *BrWAX2* gene was identified by map-based cloning and plays a role in the biosynthesis of cuticular wax. The glossy phenotype observed on the stem and leaf surface was a result of the dysfunction of *BrWAX2*, as well as the reduction of other gene expressions involved in the alkane-forming pathway [59].

Furthermore, a Chinese cabbage novel glossy mutant, known as SD369, has been discovered. The abundance of wax monomers with a carbon chain length exceeding 26 was dramatically reduced. Analysis of inheritance indicated that a single recessive gene, *BrWAX3*, controlled the glossy characteristic of SD369. The annotated genome of Chinese cabbage revealed the identification of an endoplasmic reticulum gene named *BrCER60*, which encodes a-ketoacyl-CoA synthase. This gene was found to be considerably downregulated in glossy plants’ aerial organs [72].

Another gene, named *BrCER2*, has been found as a candidate responsible for the glossy appearance of Chinese cabbage, similar to *BrCER1* and *BrCER60*. The bioinformatics analysis discovered an insertion of a long interspersed nuclear element 1 (LINE-1) transposable element (BrLINE1-RUP) in the first exon of *BrCER2*, resulting in a premature termination codon. A mutation in *BrCER2* in Chinese cabbage that impairs its function causes a significant reduction in the levels of very long chain fatty acids. This, in turn, leads to the development of a glossy pistil, cauline leaf, and inflorescence stem, as well as flower [52].

### 3.2. Vernalization

Chinese cabbage requires vernalization for flowering, and the production of haploid and doubled haploid plants is used as a biotechnological tool to shorten the breeding process for new cultivars. Doubled haploid plants possess homozygosity at all loci and can serve as parental organisms for F1 hybrids through the utilization of androgenesis induced by isolated microspore cultures [100]. Reference [100] also confirmed significant genotypic influences on the haploid embryogenesis and plant regeneration capacity in isolated microspore culture. This highlights a critical need for selecting appropriate donor material to induce androgenesis. Both haploid and diploid plants are also useful for genetic research, mutagenesis, and genetic mapping procedures. Haploidization, particularly by androgenesis, is a commonly employed technique to expedite the breeding process, which is highly advantageous in the development of novel Chinese cabbage varieties.

Chinese cabbage cultivars predominantly consist of F1 hybrids with vegetative heterosis, which are harvested using self-incompatibility or cytoplasmic male sterility for commercial seed harvesting [11]. Cytoplasmic male sterility (CMS) is a maternally inherited trait in higher plants, causing aborted or infertile pollen grains. It is crucial for heterosis utilization in Chinese cabbage and hybrid seed production, primarily caused by mitochondrial genome mutations, not nuclear gene mutations [70].

The *BrABCG26* gene is responsible for male sterility, with mutants displaying degenerated stamen and no pollen. Mutations in the second and sixth exons of the *BrABCG26* gene resulted in a loss-of-function truncated protein, affecting stamen development and reduced expression in flower buds and anthers [70]. The msm1-1, msm1-2, and msm1-3 are three allelic male-sterile mutants triggered by the same nuclear gene and had microspores aborted and abnormal tapetums. Analysis using MutMap-based gene mapping and KASP revealed that three distinct SNPSs of *BraA09g012710.3C* (*BrACOS5*) were identified as the cause of male sterility. *BrACOS5* encodes an acyl-CoA synthetase involved in the production of sporopollenin and is particularly expressed in the anther. The acyl-CoA synthetase encodes the key enzyme sporopollenin biosynthesis. Consequently, the mutation of *BrACOS5* resulted in a reduction of enzyme activities and increased the content of fatty acid in mutants anther, resulting in male sterile mutants [101].

The FLOWERING LOCUS C (FLC) genes are a key repressor of flowering in Brassica rapa, with overexpression of natural antisense transcripts (NATs) significantly shortening plant growth cycles. The sequence and expression level variation of *BrFLC* genes is extensive. A non-required vernalization in a long NAT of *BrFLC2* overexpressing lines reduced growth cycles [102]. The double knockout lines of *BraFLC2* and *BraFLC3* show an early-flowering phenotype which is vernalization-free [63]. The FLOWERING LOCUS T (FT)-like clade of phosphatidyl ethanolamine binding proteins (PEBPs) associate with the basic leucine zipper (bZIP) TF FD to induce flowering and form complexes regulatory. *BrFT1/2* promotes flowering by upregulating floral meristem identity genes in long- or short-day photoperiods [64]. Identified genes related to flowering are presented in Table 4.

### 3.3. Leafy Head and Leaf Size

Chinese cabbage has specialized leaf morphogenesis, named heading, which affects yield [73]. The leafy head formation is a specific process of leaf development that involves the growth of incurving leaves. Originating from non-heading plants around 6000 years ago, Chinese cabbage has two heading types: wrapped-over and joined-up. The degree of compactness of these Chinese cabbage varieties varies and they can be classified into loose, semi-heading, and fully-heading forms [105]. Wrapped-over refers to a plant that matures early, has a round-shaped head, and is well-suited for warmer regions. On the other hand, joined-up refers to a plant that matures later, has a longer-shaped head, and is fitted to cooler temperatures [11].

Leaf adaxial–abaxial polarity in Chinese cabbage is closely linked to leaf incurvature, which is essential for the formation of leafy heads. By identifying the specific genes that determine leaf ad-ab polarity and analysing their genetic variation, we can gain a better understanding of the underlying mechanisms behind the creation of leafy heads [31]. Leaf axial growth is also crucial during the heading stage, with no genes controlling leaf axial growth in Chinese cabbage. A previous study examined the characteristics of the mutant ic1, which exhibits inward curling, and identified *BrOPS* mutation. *BrOPS* controls the bending of leaves in a way that depends on the presence of brassinosteroids (BR). *BrOPS* and *BrBIN2* interact to regulate the phosphorylation of *BrBES1*, which in turn negatively controls the expression of the leaf polarity *BrAS1* TF. This interaction ultimately affects the curvature of leaves and the form of the heading in Chinese cabbage [73].

**Figure 2:**
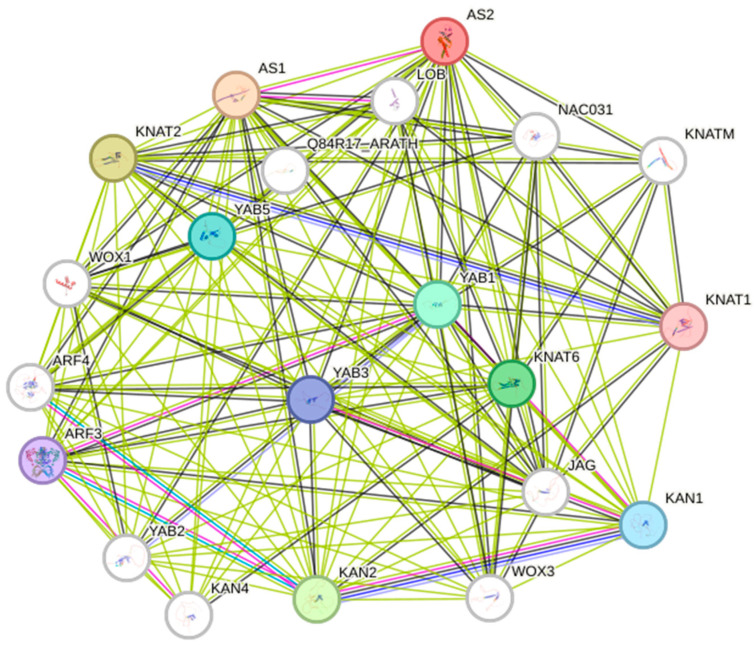
Interaction network analysis of genes related to leafy head formation in Chinese cabbage by using STRING. The interaction network has significantly more interactions than expected. This means that 21 *A. thaliana* proteins have more interactions among themselves, indicating the proteins are at least partially biologically connected as a group. The line colour is related to the type of interaction. The green line shows gene neighbourhood, the pink line means experimentally determined, the black line presents co-expression, the dark blue line indicates gene co-occurrence, and the blue line indicates protein homology. (For more interpretation of the colour codes in this figure legend, the reader is referred to the web version of this network analysis (https://string-db.org/cgi/network?taskId=bZ8uTOvikcqY&sessionId=bYgYPvAH4fiB (accessed: 30 September 2024)).

Hormones play a crucial role in the heading stage of yellow inner-leaf Chinese cabbage, affecting the expression levels of genes related to hormones like gibberellin, auxin, ethylene, cytokinin, BR, and abscisic acid, indicating the essential involvement of hormones in the heading stage [78]. Reference [78] also confirmed that several hormone metabolic pathways were identified as crucial to the developing process of leaves during the heading stage. Several differentially expressed genes (DEGs) were identified as participating in the hormonal pathways, including ko04075, ko00904, and ko00270. One group of genes exhibited differential expression in hormone biosynthesis and signal transduction pathways. As shown in Figure 2, auxin response factors (ARFs) interact with other leafy head genes (*WOX1*, *KAN2*, *AS1/2*, and so on). A previous study implied that BrAS1 correlates with BR in leafy head transformation on Chinese cabbage [73]. The transition to leaf heading is specialized leaf morphogenesis, but the transcriptional regulation mechanisms remain unclear. The ethylene pathway is particularly active in this stage, with upregulated auxin genes (*BrPIN3s*, *BrPIN4*, and *BrPIN7*) co-expressed with BR and ethylene pathways. This hormonal crosstalk may control PIN-related polar auxin transport. The study also identified a cluster of abaxial genes, including *BrEINs*, *BrARFs*, and *BrBIN2s*, interacting with hormones, the MAPK signalling pathway, microtubule dynamics, and the cytoskeleton. The cohesion among co-expression clusters offers novel insights into the interaction of distinct biological processes and suggests that ethylene, BR, and auxin might collaborate to regulate leafy head development [76].

Chinese cabbage plants have been found to have leafy heads due to an intricate hormone signalling network and abaxial–adaxial patterning pathways. Reference [74] discovered nhm4-1 and nhm4-2, two allelic non-heading mutants, and *BraA09g001440.3C* are responsible for mutant phenotypes. The concentration of gibberellic acid (GA) in the mutant leaves was noticeably decreased, and there was a tendency for the leaves to cluster together and form leafy heads after being treated with external GA_3_. This indicates that gibberellin is also involved in the development of leafy heads in Chinese cabbage [74].

Leaf flattening is crucial for plant architecture and photosynthesis. An EMS-induced population yielded a non-heading mutant with flat-heading leaves (fg-1). Phytohormone analysis showed decreased IAA, ABA, JA, SA, and increased methyl IAA and trans-Zeatin levels. Transcriptome analysis showed decreased expression levels of *BrAUX1*, *BrLAXs*, and *BrPINs*, auxin transport, and responsive genes. Furthermore, the mutation also caused an up-regulated ABA-responsive gene transcript level. A significant reduction in BrIAMT1 transcripts might contribute to leaf epinastic growth. The expression levels of crucial ABA-responsive genes, such as *BrABF3*, were increased in the middle portions of the leaves, indicating that both auxin and ABA signalling pathways have significant functions in the regulation of leaf heading. We assumed that leaf heading in Chinese cabbage is controlled through hormone signalling and abaxial–adaxial patterning pathways as previously mentioned by [71]. Furthermore, a research study discovered that a mutant cwm, characterized by its compact and wrinkled leaves, was regulated by a solitary nuclear gene known as *Brcwm*. A single SNP (on exon 4 of *BraA07g021970.3C*) resulted in a proline to serine amino acid substitution. *BraA07g021970.3C* is a homologue of *AT3G55000*, which regulates a protein involved in the regulation of cortical microtubule organization [106]. The representative candidate genes related to leafy head formation in Chinese cabbage are presented in Table 5.

Moreover, the size of leaves is essential for the growth and production of plants, particularly in Brassica crops. The control of cell division and growth in leaves is determined by a combination of signals from both the internal regulatory network and the external environment. Gaining insight into the molecular mechanism of leaf size regulation is crucial for molecular breeding aimed at enhancing agricultural productivity. Recent research has discovered multiple genes and QTLs that play a crucial role in regulating leaf size [107]. These genes regulate growth by affecting pathways such as phytohormone production [108], transcription regulation, and short RNAs [107]. The primary contributors to leaf size in Chinese cabbage are cyclins *BraA09g010980.3C* (CYCB) and *BraA10g027420.3C* (CYCD). In addition, the *BraA09gMYB47* and *BraA06gMYB88* TFs are essential in determining the leaf size difference. However, the key regulators which regulate leaf size are phytohormone homeostasis or signalling. The cell numbers influence leaf size, with GA_1_ and GA_3_ playing crucial functions in early leaf growth, while ABA and IPA regulate cell proliferation throughout leaf growth [77]. Moreover, variations in light, ambient conditions, growth factors, and DELLA proteins influence the pace at which differentiated cells elongate during the cell division phase. Longer photoperiods promote the production of GA, leading to increased expression of the important GA synthesis genes GA20ox and GA3ox, thereby stimulating the production of active GAs in Arabidopsis [109]. While in peanut, *AhGA20ox* and *AhGA3ox* function in pod growth and development [110].

**Table 5:** Candidate genes for leafy head development in Chinese cabbage.

No	Gene Name	Function	Position	References
1	*BrETR2*, *BrERS2s*, *BrEIN3s*, *BrEBFs*, *BrEILs*, *BrACSs*, *BrACOs*, and *BrERFs*	leafy head development	-	[76]
2	*REV*, *PHB*, *PHV*, *ATHB8*, *AS1*, *AS2*	leaf ad-ab polarity controlling genes	adaxial determinants	[31]
	*YAB1*, *YAB2*, *YAB3*, *YAB5*, *WOX1*, and *WOX3*		middle domain determinants	
	*KAN1*, *KAN2*, *KAN3*, *ARF3*, and *ARF4*		abaxial determinants	
	*AGO1*, *AGO7*, *AGO10*, *SGS3*, *RDR6*, *HYL1*, *DCL1*, and *DCL4*,		small RNA pathway	
3	*BrCPS1*	non-heading trait		[74]
4	*BrOPS* *BrBIN2* *BrBES1* *BrAS1*	inward curling leaves		[73]
5	*BrCWM*	compact and wrinkled leaves		[106]
6	*BrCLV1*	non-heading trait		[111]

### 3.4. Leaf Colour

Leaf colour is crucial in crucifera crops, influencing nutritional and vitamin content. Chinese cabbage has various colours like pale green, green, and orange. Studies have explored how leaf colour affects indolic glucosinolates and pigment content [23], biomass [55], flavonoid biosynthesis [112], and soluble sugar and glucosinolate biosynthesis [79].

Chlorophyll, also known as Chl, plays a vital role in the process of plant greening and is influenced by a variety of genes. The yellowing of the inner leaves of Chinese cabbage is caused by the activation of the β-carotene production pathway, which involves the upregulation of genes such as GGPP and PSY, and the downregulation of genes such as *CrtL-e*, *NCED4*, and *DWARF-27*. Several genes involved in the synthesis of chlorophyll, including *NR*, *NIR*, *GS*, *GOCAT*, and *PROA*, were identified as being downregulated [78]. Chinese cabbage leaf yellowing is linked to chlorophyll metabolism and photosystem, with the enzyme *BrHISN2*, causing it. The *CBF2-BrHISN2* module enhances gene–environment interaction for breeding [57]. A mutation in pale green mutant (pgm) caused destructive chloroplast structure resulting in lower Chl content, higher Chl a/b ratio, and lower non-photochemical quenching. Genetic analysis reveals that the pale green phenotype is controlled by a recessive nuclear gene, *BrCAO*, which upregulates *BrCAO* expression and decreases CAO enzyme activity [55].

A yellow colour in Chinese cabbage seeds can be observed from seed stages, with brown-seeded and yellow-seeded heading varieties exhibiting different patterns. It was found that brown-seeded plants have higher phenolic compounds content than yellow-seeded plants, with large fragment deletion variations in the mapping region between brown-seeded and yellow-seeded. Proanthocyanidin (PA) is the main flavonoid affecting the colour of the seed coat. The TRANSPARENT TESTA GLABRA 1 (TTG1) is the candidate gene regulating seed coat colour due to large fragment deletions in the mapping region between the two varieties. A functional marker, Brsc-yettg1, was developed to detect TTG1 variation. However, *BrTTG1* is not tissue-specific and expression levels of four structural genes were higher in brown-seeded heading than yellow-seeded heading. The levels of expression of four structural genes, namely *BrDFR*, *BrANS*, *BrANR1*, and *BrANR2*, were higher in brown-seeded heading compared to yellow-seeded heading [113]. The seed coat colour of brown-seeded seeds varied from pale green to full brown at 32 DAF, while yellow-seeded seeds showed a transition from pale green to yellow at 26 DAF. Identified genes related to leaf colour are presented in Table 6.

Flavonols and anthocyanins are the two major classes of flavonoids in Brassica rapa. The green variety of Chinese cabbage accumulates lower levels of quercetin, isorhamnetin, and cyanidin than the purple variety, illustrating that 3′-dihydroxylated flavonoids are not prevalent in the green variety. The majority of phenylpropanoid pathway gene expression patterns could not align with the patterns of flavonoid accumulation in both types (*BrPAL*, *BrC4H*, and *Br4CL*), with the exception of *BrPAL1.2*. However, it was observed that most early and late flavonoid biosynthesis genes exhibited significant levels of expression in the purple variety. Structural flavonoid metabolic profile features of purple Chinese cabbage are determined by the main enzyme, namely BrF3′H. The study also discovered that identical amino acid sequences of *BrF3′H*, which were obtained from two varieties, are highly conserved with F3′H genes from other species [112].

### 3.5. Biotic and Abiotic Stresses

Before the omics era, research conducted in the last twenty years has primarily examined the bacterial population, pesticide residues, parasite eggs, and heavy metals in Chinese cabbage due to food safety [114]. Chinese cabbage production is at risk due to abiotic and biotic stressors, climate change, and the proliferation of diseases. Research about glucosinolates (GSs) biosynthesis has been conducted in recent years [86]. GSs play a crucial role in plant–pathogen interactions and are a major secondary metabolite of the Brassicaceae family [85]. In addition, GSs and their hydrolysis products are recognized as crucial components in the interactions between plants and insects, as well as between plants and microorganisms [115]. Recent research has indicated that higher accumulation of GSs in the *BrMYC2* OE line confers the highest resistance to *S. sclerotiorum*. BrMYC2 decreased the lengths of roots and hypocotyls, seed length, and weight of Chinese cabbage. Additionally, it led to a faster bolting period and a much greater accumulation of total GSs. Elevated levels of GS accumulation in the BrMYC2OE line resulted in the greatest resistance to *S. sclerotiorum*, suggesting that overexpression may improve plant growth and development by increasing plant resistance to the fungal pathogen [85]. *Alternaria brassicifolii*, a unique species with weak pathogenicity, has been identified in *B. rapa* [116]. In addition, a first report of *Curvularia lunata* was found to cause leaf spot in Chinese cabbage [25].

Soft rot caused by *Pectobacterium carotovorum* and *Pectobacterium brasiliense* [80], and downy mildew caused by *Hyaloperonospora brassicae* [117], are the top three economically significant illnesses affecting Chinese cabbage, resulting in significant production and financial losses [118]. Polygalacturonase, pectate lyase, and cellulase are plant cell wall-degrading enzymes that are produced and released from the bacterial cytoplasm into the intercellular spaces of the plant tissue when *P. carotovorum* invades the host plant [119]. The symptoms of soft root in Chinese cabbage are characterized by a soft and watery texture, and a tan-coloured appearance [80].

A candidate pathogen-resistant WAK gene, BrWAK1, was identified and characterized, interacting with BrBAK1 to activate the mitogen-activated protein kinase (MAPK) cascade. The WAK gene, which provides disease resistance in Chinese cabbage, has been found for the first time, and its presence does not significantly influence plant biomass [117].

*Plasmodiophora brassicae* Woronin, an obligate parasite, is a major cause of clubroot, a soil-borne disease in Chinese cabbage [120], threatening the economic value of cruciferous crops [22,121]. MicroRNAs play a crucial role in post-transcriptional gene expression regulation. A total of 14 known and 10 potentially novel miRNAs were differentially expressed after *P. brassicae* treatment, with target genes mainly involved in seleno compound metabolism and plant hormone signal transduction [46]. Resistance genes to clubroot disease in *B. rapa* are located on different chromosomes. The Arabidopsis mutant rpp1 was more susceptible to clubroot disease, suggesting that deletion of rpp1 reduces plant resistance. *BrRPP1* interacts with *BrPLA1* to mediate the jasmonic acid signal transduction pathway in response to P. brassicae infection [121]. Interestingly, a study found that the causal agents of clubroot infection in *B. rapa* are robust effector-triggered immunity (ETI) and GA, as well as SA signalling [80]. Furthermore, *Variovorax* sp. YNA59 and *Lysinibacillus capsici* TT41 have been found to improve drought stress tolerance in Chinese cabbage. *Variovorax* sp. increased physiological parameters [122], while *L. capsici* enhanced drought stress tolerance by enhancing lactic acid levels [6].

Climate change has led to increased variable weather patterns affecting plants, with TFs and hormones involved in diverse plant reactions to stresses. Ethylene-responsive factors (ERFs) and ethylene or methyl jasmonate are involved in these reactions. *BrERF4* OE line improved tolerance to salt and drought stresses in Arabidopsis [83]. Reference [83] stated the *BrERF4* OE line showed green leaves after 4 days while WT showed more yellowing and began to die after 7 days of exposure to 300 mM NaCl. The study also suggests that reducing AtERF4 expression can improve salt and drought tolerance in BrERF4 OE lines by suppressing the signalling pathway mediated by AtERF4. This is supported by AtERF4’s transcriptional repressor role, resulting in increased sensitivity to abiotic stresses and the down-regulation of salt-responsive genes. High similarity is found in the genetic structure of each subfamily (Pak-choi, Chinese cabbage, and Arabidopsis), with all CSP proteins containing highly conserved domains and HSE, which respond to some abiotic stress. Additionally, each *AtCSP1/2/4/5* have HSE, which responds to some abiotic stress [53]. Interestingly, numerous BrGRP genes exhibited abnormal expression in response to multiple stresses, suggesting potential resistance to multiple stresses (Table 7) [123].

miR168a may regulate OMT1, an essential melatonin biosynthesis gene in *B. rapa*, during salt stress. This regulates melatonin content, adjusting B. rapa’s antioxidative potential, controlling osmolyte accumulation, and maintaining ion homeostasis, to improve salinity tolerance [58]. Under cold stress, a study on Chinese cabbage found that the C-repeat binding factor (CBF) response pathway could not be the primary mechanism for cold adaptation in the late-heading stage of deep yellow inner leaves. The study also found no significant differences in the expression of cold-response genes, suggesting that the CBF cold-response course is not the primary mechanism [78]. Abiotic stress, specifically Ca_2_^+^ deficient stress, causes tipburn, a collapse and necrosis of leaves. A candidate gene for tipburn resistance is *BrCRT2*, which showed higher expression levels during disease development [51].

Hydropriming boosts seed germination and early seedling growth in Chinese cabbage grown under salt stress by allowing seeds to absorb water without radicle appearance, promoting POD and CAT activities, and reducing MDA concentrations [8]. Similar to hydropriming, Nitrogen (N) is also crucial for seed germination, and deficiency is a significant environmental factor that leads to early senescence. Cotyledons provide an important N source during germination and early seedling development. Cotyledons are crucial for nitrogen during germination and seedling development. N deficiency enhances purine catabolism gene expression in Chinese cabbage cotyledons. Allopurinol, an inhibitor of xanthine dehydrogenase, reduces chlorophyll degradation and lowers fresh weight in seedlings [124]. Unfortunately, few studies have identified low N concentrations in Chinese cabbage. Nitrate transporters (NRTs) are essential for plant growth and development. The BrNRT1 gene family has a conserved structural pattern, with some genes’ expression patterns specific to tissues. The promoters of BrNRT1 contain MFS elements, indicating the potential role of *BrNRT1* in NO_3_^−^ transport regulation of Chinese cabbage [45].

**Table 7:** A (biotic) stress-related genes in Chinese cabbage.

No	Stress Type	Treatment	Gene Name	Reference
1	abiotic	low nitrate	*BrNRTs*	[45]
2	abiotic	drought stress		[122]
3	abiotic	heat stress		[9]
4	abiotic	NH_3_ emission		[125]
5	abiotic	salinity stress		[126]
6	abiotic	drought and salt stress	*BrERF4*	[83]
7	abiotic and biotic	cold, heat stress and wound stress	*BrMYB28.3* and *BrMYB29.1*	[86]
8	abiotic	salt stress	*miR168a*; *OMT1*	[58]
9	abiotic	Ca^2+^ deficiency	*BrCRT2*	[51]
10	abiotic	cold stress	*BrCSPs*	[53]
11	abiotic	freezing stress cold stress	*BrCRG1*, *BrCRG 2*, *BrCRG 3* and *BrCRG 5* *BrCRG4* and *BrCRG6*	[127]
12	abiotic	calcium deficiency, Cd stress, salt stress	*BrP450s*	[5]
13	abiotic and biotic	high and low temperature, drought, and soft rot	*BrGRPs*	[123]
14	biotic	*P. brassicae*	*BrCRa*	[120]
15	biotic	*P. brassicae*	*BrRPP1*	[121]
16	biotic	*P. brassicae*	*Bra013275*, *Bra013299*, *Bra013336*, *Bra013339*, *Bra013341*, and *Bra013357*	[50]
17	biotic	*P. brassicae*	*miRNAs*	[46]
18	biotic	*Pectobacterium carotovorum*	*WRKY38*	[34]
19	biotic	*Hyaloperonospora brassicae*	*BrWAK1*	[117]
20	biotic	*Pseudomonas syringae*	*BrPR4*	[128]

## 4. Conclusions and Future Perspective

Multi-omics analysis plays a vital role in the identification of genetic processes, growth, development, and stress tolerance in Chinese cabbage. Diverse omics methodologies analyse gene networks and molecular pathways. Despite the development of several multi-omics studies on Chinese cabbage, several key traits remain underexplored such as leaf colour, glossy leaf, and especially nutrient efficiency. The pan-omics platform, which incorporates many omics approaches, can accurately forecast agriculturally significant characteristics for precise breeding and provide insights into the molecular regulatory networks involved in enhancing Chinese cabbage. The introduction of whole genome sequencing and other breeding techniques such as SNP, MAS, and KASP has facilitated the discovery of new regulators through mutant analysis and genome-wide identification, which is a novel approach in Chinese cabbage research. These techniques are expected to advance rapidly in the field of functional genomics in the future. In addition, the utilization of the efficient transformation technique in conjunction with gene editing using CRISPR/Cas9 will result in a reduction in time required and facilitate a more precise understanding of the role of the regulators. Integrating the regulatory networks of multiple discovered characteristics in Chinese cabbage is currently a significant concern. Thus, the advancement of molecular breeding techniques has enabled the exploration of features through the selection or manipulation of specific genes. High yield, resistance to stress, delectable flavour, and affordable price will all be achieved in the end.

## Figures and Tables

**Table 1:** Representative techniques and trait improvements in Chinese cabbage genomic studies.

No	Technique	Trait Improvement	Gene Name	Reference
1	Bulk Segregant Analysis Sequencing (BSA-Seq) QTLmapping	*P. brassicae* resistance	Bra013275, Bra013299, Bra013336, Bra013339, Bra013341, and Bra013357	[50]
2	small RNA sequencing, degradome sequencing, and transcriptome sequencing	*P. brassicae resistance*	miRNAs	[46]
3	microarray	cytoplasmic male sterility	-	[35]
4	microarray		*BrMYBs*	[39]
5	Illumina HiSeq 2000 platform	male sterility	RALF genes	[56]
6	DNA-BSA, RNA-BSR, and QTL mapping	leaf yellowing	*BrHISN2*	[57]
7	computationally mined EST libraries	salt tolerance	miR168a and O-METHYLTRANSFERASE 1 (OMT1)	[58]
8	ONT ultralong-read sequencing and Hi-C technologies	evolution of centromeres	-	[3]
9	Genotyping, GWA mapping, bin-map construction, QTL analysis, BSA library construction and high-throughput DNA sequencing	-	*BrCRT2*, calreticulin gene	[51]
10	dCAPS and KASP	TMV resistance	TMV resistance gene	[37]
11	BSR-Seq, linkage analysis, and whole-genome resequencing	Pale green leaves color	*BraA10g007770.3C* (*BrCAO*)	[55]
12	BSA-Seq and KASP assays	Glossy trait	*BrWAX2*	[59]

Note: DNA-bulked segregant analysis (BSA), RNA-sequencing bulked segregant analysis (BSR), quantitative trait locus (QTL) mapping, Expressed Sequence Tag (EST), Oxford Nanopore Technology (ONT), derived cleaved amplified polymorphism sequence (dCAPS), Kompetitive Allele-Specific PCR (KASP), bulked segregant sequencing (BSA-Seq), Turnip mosaic virus (TMV).

**Table 2:** Genome editing using CRISPR/Cas, transformation method, and the Agrobacterium strain that is used in Chinese cabbage.

No.	Gene Modified	Function	Agronomic Trait	Transformation Method	Agrobacterium Strain	References
1	*BraA1000785*	unknown function	unknown agronomic trait	Agrobacterium mediated transformation	GV3101; EHA105; LB4404; AGL1	[62]
2	*BrVRN1*	delayed flowering time	flowering time	Agrobacterium mediated transformation	GV3101	[60]
3	*BraFLC*	early flowering time	flowering time	Agrobacterium mediated transformation	LB4404	[63]
4	*BrFT1*; *BrFT2*	flowering	inflorescence; flowering	Agrobacterium mediated transformation	GV3101	[64]
5	*eIF(iso)4E*	TuMV resistance	TuMV resistance	Agrobacterium mediated transformation	GV3101	[66]
6	*BrLEAFY*	bolting time	bolting time	Agrobacterium mediated transformation	LB4404	[67]

**Table 3:** Identified TFs and TF functions in Chinese cabbage.

No	TF Family	TF Names	Functions	Reference
1	MYB	*BrMYB28-1* *BrMYB28-2* *BrMYB28-3* *BrMYB29* *BrMYB29-1* *BrMYB34-1* *BrMYB34-2* *BrMYB34-3* *BrMYB34-4* *BrMYB51-1* *BrMYB34-2* *BrMYB34-3* *BrMYB122-1* *BrMYB122-2*	glucosinolate pathway	[84]
2	MYB	*BrMYB28* *BrMYB29*	glucosinolate pathway, temperature stresses	[86]
3	R2R3-MYB bHLH	*BrMYB90* *Bra037887*	anthocyanin biosynthesis	[17]
	AP2/ERF	*BrERF4*	drought and salt stress	[83]
	WRKY	*WRKY38*	pathogen	
4	MYB	*BrMYB1R25*, *BrMYB1R44*, *BrMYB1R70*, *BrMYB1R77*, *BrMYB1R171*, and *BrMYB1R 178*	cold stress	[39]
		*BrMYB222*, *BrMYB118* and *BrMYB196*	salt stress	
		*BrMYB118*, *BrMYB55*, *BrMYB168*, and *BrMYB224*	drought stress	
		*BrMYB7*, *BrMYB10*, *BrMYB55*, *BrMYB81*, *BrMYB98*, *BrMYB118*, *BrMYB168*, *BrMYB169*, *BrMYB196*, *BrMYB222*, and *BrMYB224*	ABA	
		*BrMYB81*, *BrMYB88*, *BrMYB98*, *Fusarium infectionBrMYB118*, *BrMYB163*, *BrMYB196*, *BrMYB200*, *BrMYB222*, *BrMYB224*, and *BrMYB237*	JA treatment	
		*BrMYB40*, *BrMYB58*, *BrMYB185*, *BrMYB118*, and *BrMYB222*	Fusarium infection	
5	bHLH, MADS, MYB, MYB related, WRKY, NAC, AP2-EREBP	-	yellow leafy head	[78]
6	MYB	*BrMYB51*	*Pectobacterium carotovorum* infection	[87]
		*BrMYB28.3*, *BrMYB51.1*, and *BrMYB122.2*	glucosinolate content	
		*BrMYB28s* and *BrMYB34.3*	cold, drought, salt, and ABA stress	
7	bHLH	*MYC2/3/4*	growth and development	[85]
		*BraA01gCHR11* and *BraA07gSCL1*	soluble sugar and GSL biosynthesis	[79]
8	MYB	*BraA09gMYB47* and *BraA06gMYB88*	cell size and proliferation	[77]

Note: Myeloblastosis (MYB).

**Table 4:** List of genes related to flowering in Chinese cabbage.

No	Gene Name	Function	References
1	*BrVIN3.1; BrFLC1*	bolting time	[103]
2	*BrCRY1; BrVIN3; BrGNC*	flowering time	[18]
3	*HA6*, *VATG3*, *VHA-E*, *AHA8*, *BrEXT4* and *BrYLS9*	pollen development	[35]
4	*Brcer1*	bolting and flowering	[38]
5	*BrFHAs*	male sterility	[47]
6	GSL genes (*BraA10g005220*, *BraA09g063900*, *BraA03g033140* and *BraA05g038460*) endo-1,3-b-glucosidase genes (*BraA04g008040*, *BraA08g009700* and *BraA05g015330*)	pollen development	[49]
6	*BrSKS13*	pollen development	[104]
7	*BrFLC2* NATs	flowering	[102]
8	*ARG7; SAUR41*, *BSK11*, *PYR1*, *auxin-induced protein 15A*	bolting time	[82]
9	*BrFT1* and *BrFT2*	flowering; and inflorescence organogenesis	[64]
10	*BraFLC2* and *BraFLC3*	flowering	[63]
11	*BrLEAFY*	bolting time	[67]
12	*BrACOS5*	male sterility	[101]

Notes: H+-ATPase 6 (HA6), vacuolar ATP synthase G3 (VATG3), vacuolar H+-ATPase subunit E isoform (VHA-E), and Arabidopsis H+-ATPase 8 (AHA8), Extensin 4 (BrEXT4) and yellow-leaf-specific gene 9 (BrYLS9), glucan synthase-like (GSL).

**Table 6:** Identified genes related to leaf colour in Chinese cabbage.

No	Gene Name	Trait	Function	References
1	*GGPP*, *PSY*, *CrtL-e*, *NCED4*, and *DWARF-27*.	yellow inner leaves	β-carotene biosynthesis pathway	[78]
2	*BrHISN2*	leaf yellowing		[57]
3	*Bra037887* and *MYB90/PAP2*	leaf purple	anthocyanin biosynthesis	[17]
4	*BrTTG1*	seed coat color	proanthocyanidin/flavonoid biosyntesis	[113]
5	*BrF3′H*	purple coloration	flavonoid biosynthesis	[112]
6	*BrCAO*	pale green leaves	pale green variation trait	[55]

## Data Availability

The original contributions presented in the study are included in the article, further inquiries can be directed to the corresponding author/s.
